# The Basic Biology of BACE1: A Key Therapeutic Target for Alzheimer’s Disease

**DOI:** 10.2174/138920207783769512

**Published:** 2007-12

**Authors:** S.L Cole, R Vassar

**Affiliations:** Department of Cell and Molecular Biology, Feinberg School of Medicine, Northwestern University, 303 E. Chicago Avenue, Chicago, IL 60611, USA

**Keywords:** Alzheimer’s, BACE1, β-secretase, Aβ, vascular disease, regulation, stress.

## Abstract

Alzheimer’s disease (AD) is an intractable, neurodegenerative disease that appears to be brought about by both genetic and non-genetic factors. The neuropathology associated with AD is complex, although amyloid plaques composed of the β-amyloid peptide (Aβ) are hallmark neuropathological lesions of AD brain. Indeed, Aβ plays an early and central role in this disease. β-site amyloid precursor protein (APP) cleaving enzyme 1 (BACE1) is the initiating enzyme in Aβ genesis and BACE1 levels are elevated under a variety of conditions. Given the strong correlation between Aβ and AD, and the elevation of BACE1 in this disease, this enzyme is a prime drug target for inhibiting Aβ production in AD. However, nine years on from the initial identification of BACE1, and despite intense research, a number of key questions regarding BACE1 remain unanswered. Indeed, drug discovery and development for AD continues to be challenging. While current AD therapies temporarily slow cognitive decline, treatments that address the underlying pathologic mechanisms of AD are completely lacking. Here we review the basic biology of BACE1. We pay special attention to recent research that has provided some answers to questions such as those involving the identification of novel BACE1 substrates, the potential causes of BACE1 elevation and the putative function of BACE1 in health and disease. Our increasing understanding of BACE1 biology should aid the development of compounds that interfere with BACE1 expression and activity and may lead to the generation of novel therapeutics for AD.

## INTRODUCTION

Several major pathological abnormalities characterize AD, including the Aβ-containing extracellular amyloid plaques, intracellular neurofibrillary tangles, neuroinflammation, neuronal dysfunction and ultimately neuron death. Mounting evidence suggests that Aβ plays a critical early role in AD pathogenesis. Familial AD (FAD) is caused by autosomal dominant mutations in either APP, on chromosome 21, or the presenilin (PS1, PS2) genes on chromosomes 14 and 1 respectively. A strong genetic correlation exists between FAD and the 42 amino acid Aβ form (Aβ42; reviewed in [1-3]). APP and PS mutations increase Aβ42 production and cause FAD with nearly 100% penetrance. Down’s syndrome (DS) patients, who are trisomic for chromosome 21 and thus have an extra copy of the APP gene, and FAD families with a duplicated APP gene locus [4] exhibit total Aβ overproduction and all develop early-onset AD. In FAD, the Aβ42 increase is present years before AD symptoms arise, suggesting that Aβ42 is likely to initiate AD pathophysiology.

FAD cases account for a small fraction of all AD cases and for the remaining ~98% of so-called sporadic or late-onset AD (LOAD) cases, the underlying cause (s) remain elusive, although specific risk factors for this disease are, at least in part, understood. Aging is the major risk factor for LOAD, though the basis of this association remains unknown. The apolipoprotein (Apo) E4 allele on chromosome 19, is also a strong risk factor for LOAD [5] and is associated with atherosclerosis. Interestingly, there is a close relationship between AD and cardiovascular disease with coronary artery disease, hypertension and hypercholesterolemia, amongst others, being significant AD risk factors [6-10]. How such risk factors impact AD risk and the putative molecular mechanisms underlying this association are the current focus of intense investigation. However, the robust association of Aβ42 overproduction with FAD argues strongly in favor of a critical role for Aβ42 in the etiology of AD, including in LOAD. Consequently, strategies to lower Aβ42 levels in the brain are anticipated to be of therapeutic benefit in AD. 

## Aβ GENERATION

Aβ peptide generation is mediated by the endoproteolysis of APP, a large, ubiquitously expressed, type 1 integral membrane protein, *via* the pro-amyloidogenic β- and γ-secretases, and the anti-amyloidogenic, α-secretase. BACE1 is the β-secretase that initiates, and is essential for, Aβ genesis by cleaving APP to form the N-terminus of Aβ at the Asp+1 residue of the Aβ sequence. This scission liberates two cleavage fragments: a secreted APP ectodomain, APPsβ and a membrane-bound carboxyl terminal fragment (CTF), C99. γ-secretase cleaves C99 to generate the C-terminus of the Aβ peptide and an APP intracellular domain (AICD). γ-secretase is tetrameric a protein complex [11] composed of PS1 or PS2 [12, 13], nicastrin [14], Aph1 and Pen2 [15, 16]. Cleavage by the γ-secretase complex is imprecise; while the majority of Aβ peptides liberated by γ-secretase activity are 40 amino acids in length (Aβ40), a small proportion end at amino acid 42 (Aβ42). Most FAD mutations affect the γ-secretase-dependent cleavage to cause excess generation of Aβ42 in FAD. In an alternative, non-amyloidogenic pathway, APP is cleaved by α-secretase, the activity of which precludes Aβ formation, as α-secretase cleavage occurs within the Aβ domain at Leu+17. α-secretase cleavage produces the secreted APPsα ectodomain, and a CTF, C83, which in turn is cleaved by γ-secretase to form the non-amyloidogenic 3kDa fragment, p3 and a second AICD. Three different proteases appear to be responsible for the α-secretase activity: TACE (TNF-α converting enzyme; [17], ADAM (a disintegrin and metalloprotease domain protein)-9 and ADAM-10 [18]. In many instances, the α- and β-secretase moieties compete for APP substrate [19, 20], and an increase in one cleavage event is coupled with a reciprocal decrease in the other.

## THE IDENTIFICATION OF BACE1 AS THE β-SECRETASE

In 1999-2000 several groups identified a novel aspartic protease, BACE1 (also known as memapsin 2; and Asp 2) [19, 21-24] as the β-secretase. BACE1 exhibited all the previously determined characteristics of β-secretase. Earlier, maximal β-secretase activity had been observed in neural tissue and neuronal cell lines [25], although some activity was detected in the majority of body tissues [26]. Interestingly, astrocytes exhibited less β-secretase activity than neurons [27]. Indeed, the pattern and level of BACE1 expression is largely consistent with those of β-secretase activity in cells and tissues [19, 23, 28]. The levels of BACE1 mRNA are highest in brain and pancreas and are significantly lower in most other tissues. Moreover, BACE1 mRNA is highly expressed in neurons but little is found in resting glial cells of the brain, as expected for β-secretase. The protein is abundant in both normal human and AD brain [19, 28]. The high pancreatic mRNA expression was initially confusing, given the low levels of β-secretase activity in this tissue [22]. However, it was subsequently reported that BACE1 mRNA transcripts in pancreas largely consist of a splice variant missing the majority of exon 3 [29, 30]. This splice variant encodes a BACE1 isoform devoid of β-secretase activity, thus reconciling the paradoxically high BACE1 mRNA levels with the low β-secretase activity found in the pancreas. The functional relevance of this pancreas-specific splice variant remains unclear.

When transfected into stable APP-overexpressing cell lines, BACE1 induces a dramatic increase in β-secretase activity. APPsβ and C99, the immediate products of β-secretase cleavage, are increased several fold over levels found in untransfected cells, and Aβ production is also elevated. Interestingly, APPsα levels are reduced upon BACE1 transfection, suggesting that α- and β-secretases compete for APP substrate in cells. Treatment of APP-overexpressing cells with BACE1 antisense oligonucleotides decreases BACE1 mRNA levels, inhibits β-secretase activity [19, 23] and reduces production of APPsβ, C99, and Aβ in cells; conversely, APPsα and C83 generation is elevated.

β-Secretase activity in cells efficiently cleaves membrane-bound substrates only [31], and has maximal activity at acidic pH, [32-34], with the highest activity being detected in the acidic subcellular compartments of the secretory pathway, including the Golgi apparatus, trans Golgi network (TGN), and endosomes [35, 36]. These data suggest that the active site of β-secretase is located within the lumen of acidic intracellular compartments. As predicted, BACE1 is resistant to inhibition by pepstatin and has optimal activity at ~pH 4.5, being localized within acidic subcellular compartments of the secretory pathway, primarily the Golgi apparatus, TGN and endosomes. The 501 amino acid sequence of BACE1 bears the hallmark features of eukaryotic aspartic proteases of the pepsin family. BACE1 has two aspartic protease active site motifs, DTGS (residues 93-96) and DSGT (residues 289-292), and mutation of either aspartic acid renders the enzyme inactive [21, 37]. Like other aspartic proteases, BACE1 has an N-terminal signal sequence (residues 1-21) and a pro-peptide domain (residues 22-45) that are removed post-translationally, so the mature enzyme begins at residue Glu46 [37]. Importantly, BACE1 has a single transmembrane domain near its C-terminus (residues 455-480) and a palmitoylated cytoplasmic tail [38]. BACE1 is a type I membrane protein with a luminal active site, features predicted for β-secretase. The position of the BACE1 active site within the lumen of intracellular compartments provides the correct topological orientation for cleavage of APP at the β-secretase site. Similar to other aspartic proteases, BACE1 has several N-linked glycosylation sites and six luminal cysteine residues that form three intramolecular disulfide bonds [39].

Site-directed mutagenesis of the amino acids surrounding the cleavage site in APP defined the sequence preference of the β-secretase [31], and the complete specificity of this protease was derived on the basis of substrate kinetics and the screening of a combinatorial inhibitor library [40]. Substitutions of larger hydrophobic amino acids (such as Leu found in the Swedish FAD mutation) for the Met residue at P1 improve the efficiency of β-secretase cleavage. Conversely, substitution of the smaller hydrophobic amino acid Val at the same position inhibits cleavage. Many other substitutions at this site and at surrounding positions decrease cleavage, and indicate that the β-secretase is highly sequence-specific. Interestingly, a number of Aβ species with different N-terminus residues have been identified. Radiosequencing demonstrated that Aβ isolated from amyloid plaques, as well as that produced in cell lines, predominantly begins at the Asp+1 residue of Aβ [41], although minor Aβ species begin at Val-3, Ile-6, and Glu+11 [26]. Inhibitor studies suggest that the Val-3 and Ile-6 species are generated by a protease that is different from the β-secretase [42]. However, the Glu+11 species is produced in parallel with Asp+1 Aβ [43], suggesting that β-secretase is responsible for cleaving at both these positions. Interestingly, the Glu+11 species is the predominant form of Aβ made in rat primary neuron cultures [43]. Importantly, BACE1 cleaves APP only at the known β-secretase sites of Asp+1 and Glu+11 of Aβ [19]. Moreover, purified recombinant BACE1 directly cleaves APP substrates at these same sites *in vitro*, demonstrating that the BACE1 molecule intrinsically exhibits protease activity [19, 23]. The sequence specificity of purified BACE1 is the same as β-secretase. For example, it cleaves Swedish mutant APP substrate much more efficiently than wild type, and does not cleave a P1 Met-Val mutant substrate that is resistant to β-secretase cleavage. 

Taken as a whole, the properties of BACE1 correlate very well with the previously established characteristics of β-secretase activity in cells and tissues.

## BACE1 AS A THERAPEUTIC TARGET

BACE1 is considered a prime drug target for lowering cerebral Aβ levels in the treatment and/or prevention of AD for a number of reasons. Most importantly, BACE1 is the initiating and putatively rate-limiting enzyme in Aβ generation and BACE1 inhibition would block the production of Aβ and prevent the development of Aβ-associated pathologies. Furthermore, initial reports indicated that BACE1 knockout (BACE1^-/-^) mice were free of any harsh phenotype and showed no pathological abnormalities, although more recent analysis does indicate subtle behavioral alterations in mice lacking BACE1 expression (discussed below). It has been speculated that BACE1 is a more suitable therapeutic target than either γ - or α-secretase, although, as described later, there are a number of caveats associated with BACE1 inhibition. Presenilin, the catalytic member of the γ-secretase complex, is involved in the Notch signaling pathway [44] and knocking out PS1 causes embryonic lethality [45]. While mice showing reduced PS expression and conditional neuronal knockouts are viable, they exhibit behavioral deficits [46, 47] and a report indicates that they may have potential for initiating tumorigenesis [48]. In the case of α-secretase, a potential treatment strategy would be to enhance the cleavage activities of this enzyme. However, such an approach is perceived to be more challenging that inhibiting cleavage by the pro-amyloidogenic secretases (reviewed in [49]).

## BACE1 KNOCKOUTS 

For the development of useful, safe BACE1 inhibitors, understanding the function (s) of BACE1 *in vivo* is critically important. Not only has data derived from BACE1^-/-^ mice provided unequivocal proof that BACE1 is the major brain β-secretase, but it has alsobeen used to determine whether BACE1 has any vital function *in vivo*, or if it is dispensable.

Initial data indicated that the lack of BACE1 expression did not appear to adversely affect embryonic development, nor did it significantly affect the morphology, physiology, biochemistry, and gross behavior of post-natal or adult knockout mice [50, 51]. Investigations of BACE1^-/- ^mice brain function established that no demonstrable differences existed, as compared to wild-type mice. Overall these early findings indicated that the absence of BACE1 is well tolerated *in vivo* and does not appear to cause untoward effects in the developing, post-natal, or adult mouse.

Data generated from BACE1-deficient mouse models was unanimous in demonstrating that β-secretase activity is abolished in brains and cultured neurons of BACE1^-/- ^mice. Unlike wild-type mice, which make low levels of endogenous Aβ, transgenic (Tg) mice overexpressing human APP with the Swedish mutation (Tg2576; [52]) produce robust levels of brain Aβ and develop Aβ plaques with age. Importantly, BACE1^-/-^•Tg2576 bigenic mice lacked all forms of Aβ, as well as APPsβ and C99, as compared to BACE1^+/-^•Tg2576 or BACE1^+/+^•Tg2576 mice [50]. Thus all products of APP processing by β-secretase, including Aβ, were abolished in BACE1^-/-^ brain, unequivocally proving that BACE1 is the major, if not only, β-secretase responsible for Aβ generation in the brain. *In vitro* data from BACE1^-/-^ primary neurons and brain tissue added further support for the predominant role of BACE1 in Aβ generation [51, 53]. BACE1^-/-^•Tg2576 mice not only lack cerebral Aβ, but also fail to develop amyloid plaques with age [54]. In contrast to Tg2576 mice that begin to deposit brain amyloid at ~9-12 months, BACE1^-/-^•Tg2576 bigenic mice show no evidence of amyloid deposits even by 13 months. Similar results were obtained using different BACE1^-/-^ and APP Tg mice [55]. These results demonstrate BACE1 is essential for amyloid formation.

Multiple Aβ species, including extracellular fibrillar Aβ, intracellular and soluble, oligomeric Aβ, accumulate in AD brain [56, 57]. We have recently conducted studies to determine whether BACE1 deficiency, and the consequent ablation of Aβ, is sufficient to rescue memory deficits in Tg2576 mice [58]. Tg2576 mice develop memory impairments at an early age before the onset of amyloid deposition [59], and we chose to study young pre-deposit mice to analyze the contribution of soluble Aβ to memory dysfunction. We demonstrated that memory deficits and cholinergic dysfunction in the hippocampus did not develop in BACE1^-/-^•Tg2576 bigenic mice that lacked Aβ, while florid deficits were apparent in Aβ-overproducing Tg2576 monogenics. At the time of testing the Aβ in Tg2576 mice was non-fibrillar and soluble, thus we concluded that soluble Aβ assemblies rather than amyloid plaques are responsible for at least some aspects of AD-related memory deficits. Our work is further validation of BACE1 as a prime therapeutic target for AD and provides direct evidence for the amyloid hypothesis *in vivo*. 

Clearly, the consequences of BACE1 ablation in aged animals, which exhibit significant amyloid pathology, should also be closely examined. To this end, studies involving the use of APP/PS1 double Tg mice, which exhibit accelerated Aβ accumulation and AD-associated memory deficits as compared to single Tg mice, have been performed. While the water maze learning was impaired in aged APPswe;PS1ΔE9 Tg mice, which display abundant amyloid pathology, APPswe;PS1ΔE9 lacking BACE1 performed as well as wild type controls [55]. Amyloid pathology was not detected in the APPswe;PS1ΔE9;BACE1^-/-^ mice, thus demonstrating that BACE1 deletion abolishes amyloid deposition and prevented spatial reference memory deficits in aged APPswe;PS1ΔE9 mice. Similar data was derived from our 5XFAD APP/PS1 Tg mouse model [60-62]. 5XFAD mice co-express an APP transgene carrying the Swedish (K670N, M671L), London (V717I) and Florida (I716V) mutations and a PS1 transgene carrying double FAD mutations (M146L and L286V; [60]. 5XFAD mice exhibit aggressive pathology with plaque deposition occurring at 2 months and, unlike many Tg models of AD, exhibit significant neuronal loss in AD-sensitive brain regions [60]. The deficits in hippocampus-dependent temporal associative learning found in 5XFAD mice were rescued in BACE1^-/-^;5XFAD mice [61] reviewed in [63]. Not only were the elevated Aβ levels observed in 5XFAD mice reduced to wild type control levels in BACE1^-/-^; 5XFAD bigenic mouse brain, but the genetic abrogation of BACE1 also prevented neuronal loss [62]. These data provide strong support for the role of Aβ peptides in age-associated cognitive impairments, and also indicate that Aβ is ultimately responsible for neuronal death, at least in Tg models of AD.

Although the initial studies of BACE1 knockout mice did not reveal gross alterations in behavior, recently, more precise behavioral phenotyping studies of BACE1^-/- ^mice have revealed abnormalities in cognitive and emotional functions, suggesting potential mechanism-based toxicities resulting from complete BACE1 inhibition [55, 58, 61]. We showed that BACE1^-/- ^mice were impaired in both spatial and reference memories. These mice also exhibited impairments in temporal associative memory although they appeared normal in social recognition. These data raise the possibility that BACE1 is required for some normal hippocampal memory processes [58, 61]. Consistent with our findings, Laird [55] reported that mice deficient in BACE1 exhibited impaired spatial reference and working memories.

In addition to the putative effect of complete BACE1 ablation on specific cognitive functions, recent findings have indicated that BACE1 deficiency might be associated with a higher mortality rate in early life [64]. BACE1^-/- ^mice also exhibited other learning-unrelated phenotypes, appearing hyperactive, with enhanced locomotion [55, 64]. BACE1^-/- ^mice spent more time in central parts of the open-field and visited open-arms in the plus-maze test more often than wild type controls, indicating that BACE1 may play some role in emotion [55]. However, the putative role that BACE1 may play in emotion appears complex. In contrast to Laird [18], Harrison and colleagues previously reported that BACE1^-/-^ mice showed timid, more anxious behavior [65]. While strain differences may account for these apparently contrasting data, the exact reasons underlying these opposing effects of BACE1 on emotion remain to be determined. 

Interestingly, Singer and colleagues demonstrated that a partial reduction in BACE1 can improve amyloid neuropathology including the deposition of Aβ, alongside cognitive deficits in APP Tg mice [66]. Indeed, comparison of BACE1 homozygous and heterozygous (BACE1^+/-^) knockout mice indicated that BACE1^+/- ^mice exhibited normal spatial memory function compared to BACE1^-/- ^mice, suggesting that partial inhibition of BACE1 may not affect normallearning and memory processes. Such information is of crucial importance given the fact that when suitable BACE1 inhibitors are developed, it will be almost impossible to completely suppress BACE1 enzymatic activity *in vivo*. In this regard, Laird demonstrated that Aβ burden appears sensitive to BACE1 dosage in young animals, with Aβ levels reduced to ~60-70% in APPswe;PS1ΔE9; BACE1^+/- ^compared to age-matched APPswe;PS1ΔE9 animals [55]. However, the Aβ burden was not altered in older mice, despite the same decrease in BACE1 levels, indicating that BACE1 is no longer a limiting factor in the aged mouse. Consistent with these findings was that older APPswe;PS1ΔE9; BACE1^+/- ^micewere significantly impaired in the Morris water maze, indicating that 50% reductions in BACE are not sufficient to significantly ameliorate cognitive deficits in aged mouse in contrast to complete BACE1 ablation. Although further work is required, these data indicate that a partial suppression of BACE1 may have the most benefit for the earlier phases of Aβ-dependent cognitive impairments. However, the age-dependent benefits of partial reduction BACE1 expression appear complicated. McConlogue and colleagues recently reported that, in contrast to the observations of Laird, a 50% decrease in BACE1 levels exerts little impact on Aβ levels in young APP Tg mice, but led to a dramatic reduction in Aβ levels and synaptic deficits in aged mice [67]. The reasons as to why these findings appear discordant with those of the previous study remain to be determined, but may be due to the expression of different APP moieties, APP with the Swedish mutation [55] *vs*. APP with the V717F mutation [67]. The Swedish mutation enhances the cleavage of APP by BACE1. As McConlogue and colleagues discuss, the co-expression of APPswe with the PS1 transgene in the Laird study led to an aggressive model of plaque development that may somehow impact the sensitivity of APP metabolism to alterations in BACE1 levels.

Despite the apparent benefits of a reduction in BACE1 levels reported above, there has been a recent study that contains data that may be a cause for some concern. While the BACE1-mediated cleavage of APP is predominantly regarded as a negative process, Ma and colleagues reported the facilitation of learning, memory and synaptic plasticity by this cleavage event [68]. In this study, APP-overproducing mice exhibited enhanced spatial memory rather than AD pathology or memory deficits. Interestingly, a significant decrease in AICD (but not of any other APP cleavage products) abolished the enhanced synaptic plasticity and memory. The authors propose that these data indicate that normal brain functions are facilitated by the regulated, activity-dependent cleavage of APP by BACE1 in neurons. Importantly, AICD levels decreased significantly in both BACE1 heterozygous and null mice and it was noted that the removal of one BACE1 gene dramatically reduced AICD without altering other APP cleavage product levels. As discussed by Ma and colleagues, these data stress the requirement for thorough preclinical studies of experimental BACE1 inhibitors, not only in animal models of AD, but also in wild type animals to evaluate their effect on normal cognitive functioning [68]. These data await confirmation from other studies.

In conclusion, the lack of Aβ generation in the brains of BACE1 deficient mice indicates that therapeutic inhibition of BACE1 should reduce cerebral Aβ levels and amyloid development, an outcome likely to be beneficial for AD. While recent studies indicate that complete blockage of BACE1 activity may be associated with certain undesirable side-effects, important data demonstrates that partial reduction of BACE1 levels may improve cognitive deficits and amyloid neuropathology including Aβ deposition, at least in specific AD Tg models. However, given that AICD may be required for normal memory, the degree of BACE1 inhibition required to have beneficial lowering effects on Aβ levels without impacting AICD levels remains to be determined.

## BACE2: MORE LIKE α- THAN β-SECRETASE

Due to redundancy, the lowering or removal of one molecule may be compensated for by the activities of another. Clearly, data from BACE1^-/-^ mice unequivocally prove that BACE1 is the β-secretase responsible for Aβ generation. However, BACE2, a BACE1 homologue, has been identified. It was plausible that knowledge of homologous BACE1 proteins may prove useful for the determination of the BACE1 function *in vivo*. Indeed, given that BACE1 mRNA [28, 69] and protein [70] are detected at high levels in brain areas that are devoid of APP and which have very low Aβ plaque counts, such as the striatum and thalamus, alternative BACE1 substrates in different brain regions may exist, as discussed later. 

BACE2 is mapped to the DS critical region on chromosome 21 [71, 72]. The amino acid sequences of BACE1 and BACE2 are ~45% identical and 75% homologous [73]. Overall the BACE2 structure contains features typical of this protease family. BACE1 and BACE2 have distinct transcriptional regulation and function [73]. BACE2 mRNA has been observed at low levels in most human peripheral tissues. However, unlike BACE1, which is enriched in neuronal populations [19], human adult and fetal whole brain express very low or undetectable levels of BACE2 mRNA [28, 74]. *In vitro*, BACE2 can cleave APP at the β-secretase cleavage site [75, 76] and BACE2 appears to be primarily responsible for Aβ production in Flemish mutant APP transfected cells [75]. However, other studies have demonstrated that BACE2 functions as an alternative α-secretase and as an antagonist of BACE1 [77, 78]. BACE2 does not get upregulated to compensate for a lack of BACE1 in knockout mice [54]. Further evidence that BACE2 functions not as a β-secretase comes from a recent study which identifies BACE2 as a novel theta (θ)-secretase. Radiosequencing clearly demonstrated that the major BACE2 cleavage site is between Phe+19 and Phe+20 sites of APP [79, 80], thus BACE2 cleaves APP at a novel q-site downstream of the α-secretase cleavage site [81]. Cleavage of APP by BACE2 at this site abolishes Aβ production. Furthermore, overexpression of BACE2 reduced Aβ production in primary neuronal cultures derived from APP transgenic (Tg) mice [81].

## BACE1 GENE

Given that the β-secretase gene is a strong candidate gene for LOAD because of its function in initiating Aβ production, and that the etiology of the disease remains unknown, the genomic organization of the BACE1 gene and the presence of polymorphisms have been closely examined. The BACE1 gene spans ~30 kilobases on human chromosome 11q23.2 and includes 9 exons. The data regarding the association between polymorphisms in BACE1 and AD were initially mixed, although more recent findings from a number of groups concur that polymorphisms in exon 5 of BACE1 are associated with AD. Murphy and colleagues [82] identified two polymorphisms in exon 5 and intron 5 of the BACE1 gene. However, neither appeared to be associated with AD risk in genetic association studies comparing late-onset AD cases (autopsy-confirmed) with age-matched non-demented controls. Nevertheless, Nowotny reported that there was a weak association between the BACE1 exon 5 polymorphism and AD in those carrying the ApoE4 allele [83]. Indeed, a later study that analyzed three polymorphisms located at the BACE1 gene, found an association between BACE1 exon 5 GG genotype and AD. The authors state that in combination with earlier results, there is a clear increase in the risk of developing AD in subjects carrying both the BACE1 exon 5 GG genotype and the ApoE4 allele [84]. Kirschling and co-workers reported that the BACE1 exon 5 polymorphism influenced AD risk and that this effect was indeed most pronounced in ApoE4 allele carriers [85]. Furthermore, while initial genotype data from a late-onset AD group and a control cohort failed to demonstrate any association between BACE1 and AD, following stratification for ApoE status it was concluded that a combination of a specific BACE1 allele (within BACE1 codon V262) and ApoE4 may increase AD risk over that attributed to ApoE4 alone [86]. A very recent meta-analysis study investigating AD association with BACE1 exon 5, demonstrated that the genotype CC+CT acts as a protective factor in ApoE4 carriers, and as a risk factor in apoE4 non-carriers [87]. To date the mechanisms that underlie the associations of BACE1 exon 5 polymorphisms with AD remain to be established. 

Importantly, a recent study has highlighted the feasibility of the ultra-high density whole-genome association approach to the study of AD. Indeed predictions are that such approaches show potential for the future identification of additional genes that contribute to AD [88].****Clearly, the ApoE4 allele is a well-established risk factor for sporadic AD. However, this locus on chromosome 19 was not identified in initial genome scans using microsatellite markers in late-onset AD. However, ultra-high density single nucleotide polymorphisms (SNP) genotyping (a method to simultaneously assess hundreds of thousands of SNPs) precisely identified the ApoE locus as being significantly associated with late-onset AD (Coon *et al*., 2007). As no other SNP showed such as robust an association these data support the indisputable notion that the epsilon 4 variant of ApoE is the biggest genetic risk factor for LOAD.

## THE CELL BIOLOGY OF BACE1 

To gain insight into the regulation of BACE1 and identify other potential therapeutic targets in the β-secretase pathway, groups began investigating the cell biology of BACE1. BACE1 is initially synthesized in the endoplasmic reticulum (ER) as an immature precursor protein (proBACE1) with a molecular mass of ~60kDa [39, 89-91]. Site-directed mutagenesis indicates that acetylation of seven lysine residues of the N-terminal portion of nascent BACE1 is required for the protein to leave the ER and to progress through the secretory pathway [92]. ProBACE1 is short-lived and undergoes rapid maturation into a 70kDa form in the Golgi, which involves the addition of complex carbohydrates and the removal of the propeptide domain. 

BACE1 is glycosylated at four N-linked sites [39], a modification which affects the protease activity of the enzyme since abolishing two out of four N-linked sites by site-directed mutagenesis significantly reduces β-secretase activity [93]. Mature N-glycosylated moieties of BACE1 are sulfated and three cysteine residues within the cytosolic tail of BACE1 are palmitoylated, which might influence intracellular localization or trafficking [38]. The BACE1 propeptide domain is removed within the Golgi apparatus by cleavage between Arg45 and Glu46 [74]. While the majority of aspartic proteases, including BACE2, cleave the propeptide domain autocatalytically in response to changes in pH or ionic strength [94], BACE1 propeptide removal involves intermolecular cleavage by a different protease. Furin, a ubiquitous Golgi-localized proprotein convertase (PC) appears to be the major protease mediating BACE1 propeptide removal, although other PCs are also capable of cleaving the BACE1 propeptide [38, 74, 91]. Unlike other zymogens, proBACE1 exhibits robust β-secretase activity, suggesting that the BACE1 propeptide domain does not suppress protease activity significantly [38, 91]. Indeed, proBACE1 may cleave APP early in the biosynthetic pathway leading to the generation of an intracellular pool of Aβ in the ER, thought by some investigators to be particularly neurotoxic [91]. Following maturation, the lysine residues are deacetylated in the Golgi lumen [92]. Mature BACE1 localizes largely within cholesterol-rich lipid rafts [95, 96], and is quite stable, having a half-life of over nine hours in cultured cells.

In cultured polarized cells such as neurons or Madin-Darby canine kidney cells, BACE1 is predominantly transported to the axonal/apical compartment, while APP and α-secretase are sorted mainly to the somatodendritic/basola-teral compartment [97]. This trafficking pattern is consistent with observations *in vivo* indicating that BACE1 is transported down axons of the perforant pathway [98] and that axon terminals may be major sites of Aβ production [98, 99]. Further data about the subcellular trafficking and localization of BACE1 is detailed later.

Although the majority of BACE1 in cells is produced as an integral membrane protein, a small fraction of BACE1 undergoes ectodomain shedding, a process that is suppressed by palmitoylation [38]. Inhibition of shedding does not influence processing of APP at the β-site [100]. However, co-expression of APP and the soluble ectodomain of BACE1 in cells increases the generation of Aβ, suggesting the enhanced BACE1 ectodomain shedding may raise amyloidogenic processing of APP [38]. Murayama and colleagues reported the release of detectable levels of BACE1 holoprotein *in vitro* [101], although the physiological relevance of this event remains to be clarified. Interestingly, active, soluble BACE1 has been detected in human cerebral spinal fluid (CSF), [102], a finding which raises the potential use of BACE1 detection in an easily accessible biological fluid, such as CSF, in diagnostic or prognostic applications in the future.

## BACE X-RAY STRUCTURE

Structural information about the interaction of substrate with the active site of BACE1 would greatly facilitate the rational design of small molecule BACE1 inhibitors. Molecular modeling was used to simulate the BACE1 active site bound with wild-type or mutant APP substrates [103], and several residues that potentially contribute to substrate specificity in BACE1 were identified. Shortly afterwards, the x-ray structure of the BACE1 protease domain co-crystallized with a transition-state inhibitor was determined to 1.9 angstrom resolution [104]. As expected, the BACE1 catalytic domain is similar in structure to pepsin and other aspartic proteases, despite the relatively low sequence similarity. Interestingly, the BACE1 active site is more open and less hydrophobic than that of other aspartic proteases. Four hydrogen bonds from the catalytic aspartic acid residues (Asp93 and Asp289) and ten additional hydrogen bonds from various residues in the active site are made with the inhibitor, most of which are conserved in other aspartic proteases. The x-ray structure indicates that Arg296 and the hydrophobic pocket of the active site play an important role in substrate binding, confirming the results of the molecular modeling study. In addition, the bound inhibitor has an unusual kinked conformation from P2’ to P4’. The kink directed residues P3’and P4’ toward the protein surface have little protein-inhibitor interaction, suggesting the absence of S3’ and S4’ subsites in BACE1. However, subsequent crystal structure of OM00-3 binding to BACE1 showed clear interaction at these two positions and thus identified the location of these two subsites on the protease [105]. The BACE1 X-ray structure suggests that small molecules targeting Arg296 and the hydrophobic pocket residues should inhibit β-secretase cleavage. Moreover, mimicking the unique P2’-P4’ conformation of the bound inhibitor may increase the selectivity of inhibitors for BACE1 over BACE2 and the other aspartic proteases.

## BACE1 SUBSTRATES

BACE1 is crucial for Aβ generation, and the normal production of Aβ in the brain raises the possibility that rather than being a toxic by-product of APP metabolism, Aβ may fulfill a regular physiological function. Indeed, our examination of BACE knockout mice indicated that complete abrogation of Aβ was associated with impaired memory performance, data suggestive of a role for Aβ in normal memory [58]. These data were consistent with earlier reports of a potential physiological role of Aβ in normal neuronal function [46, 106]. Indeed, a recent report suggests that the production of endogenous Aβ is an important physiological regulator of potassium channel expression and negatively modulates neuronal excitability [107]. However, our data indicate that BACE1 deficiency does not impact all types of hippocampal learning [58] and it is clear that further work is required to examine the putative normal role of Aβ *in vivo*, under non-pathological conditions.

In addition to APP, BACE1 also cleaves the APP homologues, amyloid precursor-like proteins, APLP1 and APLP2 [108, 109]. While APLP1 and APLP2 lack the Aβ sequence, relatively little else is known about these proteins, although it is known that the APLPs can be processed by γ-secretase generating intracellular fragments with potential transcriptional activity [110, 111].

Given the fact that the majority of APP molecules are cleaved by α-secretase moieties, with only a small fraction being BACE1 substrate, it is highly likely that other BACE1 substrates exist. While the essential cellular functions of BACE1 under normal conditions have proved somewhat elusive, recent findings have started to define a physiological function for this enzyme. 

BACE1 is enriched in neuronal populations, and β-secretase processing of APP is modulated by the interaction of BACE1 with neurite growth inhibitor NOGO, a component of myelin, and a member of the reticulon family of proteins [112]. Indeed, BACE1 interacts with all members of the reticulon protein family [112, 113]. Importantly, a role for BACE1 in axonal growth and brain development has been recently proposed, whereby BACE1 regulates the myelination process in both peripheral and central nerves [114, 115]. The neuronal type III isoform of the epidermal growth factor (EGF)-like factor neuregulin 1 (NRG1) regulates myelination and is a known initiator of peripheral nervous system (PNS) myelination and a modulator of myelin sheath thickness in both the central nervous system (CNS) and the PNS. BACE1 is transported to axons by a kinesin-1-dependent pathway [116] and the highest levels of BACE1 expression are observed when myelination occurs during the early postnatal stages [115]. In situ hybridization revealed co-expression of BACE1 with type III NRG1 within sensory and motor neurons whose axons project within peripheral nerves. Importantly, data generated from BACE1 null mice showed that the absence of BACE1 was associated with hypomyelination of both CNS and PNS axons. 

Interestingly, previous reports indicated that mice deficient in BACE1 exhibit altered hippocampal synaptic plasticity and decreased cognitive function [55, 58, 61] reviewed in [63]. Indeed, specific neurological impairments were associated with the genetic ablation of BACE1 in this myelination study [114]. At the molecular level, BACE1^-/-^ brain, when compared to wild type brain, accumulated uncleaved NRG1 and exhibited reduced levels of NRG1 cleavage fragments, findings consistent with a role for BACE1 in the proteolysis of NRG1 [114, 115]. It should be noted that complete deletion of BACE1 was necessary in order to alter the signaling events and cause hypomyelination. In agreement with some (but not all [68]) previous findings that partial BACE1 inhibition may be without effect on normal learning and memory processes, [55] reviewed in [63], BACE^+/-^ mice did not display any of the biochemical and morphological features observed in the BACE1 null mice. 

It is known that synaptic dysfunction precedes overt neurodegeneration in AD development. Research supports the notion that BACE1 cleaves APP to generate Aβ in the synaptic terminal [98, 99]. Several additional, non-APP BACE1 substrates have recently been identified, some of which may be localized at the terminal, suggesting that BACE1 cleavage of particular substrates may be required for normal function at the synapse. 

Voltage-gated sodium channels (VGSC; Na_v_) are abundant ion channels responsible for the initiation and propagation of action potentials. These channels are complexes consisting of α and β subunits. Although not essential for the basic VGSC functioning, the VGSCβ subunits are important auxiliary units. Expression of both subunit types is required for full VGSC functionality. Importantly, VGSCβ subunits are BACE1 substrates, and BACE1 cleavage generates a membrane-bound β-CTF [117, 118]. Furthermore, analogous to APP processing, VGSCβ subunits are processed further by γ-secretase, which generates an β2-intracellular domain (β2-ICD; [118]). The functional ramifications of these cleavage events have been recently elucidated. β2-ICD regulates expression of the α subunit, Na_v_1.1. Importantly, the increased pool of Na_v_1.1 is maintained within the cell and the BACE1 cleavage of the β2 subunit actually leads to loss of functional membrane channels, a reduction in Na^+^ current and alterations in membrane excitability [118]. Importantly, the processing of VGSCβ2 and VGSCβ4 by BACE1 has been demonstrated *in vivo* and elevated β2-CTF and Na_v_1.1 were observed in human AD brain tissue [117, 118]. Neural activity regulates Aβ production through β- and γ-secretase and synaptic transmission and neuronal activity is depressed by Aβ [106, 119]. It is thus plausible that the turnover of membrane-localized functional sodium channels by the sequential processing by BACE1 and γ-secretase in wild type neurons may play a role in such a feedback mechanism as to modulate neuronal activity and endogenous Aβ production. In addition, processing of VGSCβ4 by BACE1 may also regulate the filopodia-like protrusion density in neurons together with neurite length [120]. 

The proteolytic processing of membrane proteins to their soluble counterparts during ectodomain shedding is an important step in regulating the biological activity of membrane proteins. Ectodomain shedding is carried out by members of the ADAM family, and to a lesser extent by BACE (reviewed in [121]). This is the first event in a two-step proteolytic cleavage event known as regulated intramembrane proteolysis (RIP). Subsequently, the resulting membrane bound CTF, undergoes a second cleavage within its transmembrane domain, called intramembrane proteolysis. APP undergoes RIP by the α- and β-secretases, with the intramembrane proteolysis being catalyzed by the γ-secretase complex. Indeed, studies have proposed that other BACE1 substrates may be found among proteins undergoing ectodomain shedding, as discussed below. 

Another putative BACE1 substrate with a proposed neuronal function, which also undergoes ectodomain shedding, is the lipoprotein receptor-related protein (LRP). Accumulating evidence indicates that elevated cholesterol might be closely involved in AD development. LRP is a type I integral membrane protein which functions as a multifunctional endocytic receptor and has been implicated as having signaling roles in neurons [122]. Furthermore, LRP appears to be intimately associated with AD pathology and previous reports have implicated LRP in the mediation of endocytosis of a number of important AD-linked ligands including APP and ApoE. Indeed, it has been shown that LRP regulates Aβ trafficking, binds Aβ complexes and mediates its degradation. *In vivo*, the absence of LRP in the presence of APP overexpression led to a two fold increase in amyloid deposition, findings supporting the notion that the LRP might play an integral role in Aβ clearance and might be neuroprotective against Aβ toxicity [123]. LRP is also processed in manner analogous to APP, at least *in vitro*. Not only is LRP proteolyzed by matrix metalloproteases, but the γ-secretase cleavage of LRP enables release of the LRP-intracellular domain (LRP-ICD). Furthermore, LRP may be a substrate for BACE1 cleavage [124]. Endogenous BACE1 and LRP co-immunoprecipitate from human brain and it appears as if the LRP-BACE1 complexes occur in lipid-rafts, with the closest association being at the cell surface. Further *in vitro* analyses demonstrated that endogenous levels of BACE1 activity facilitated an increase in the secretion of LRP, in addition to the formation of the LRP CTF. Moreover, increased BACE1 expression facilitated an enhancement of γ-secretase LRP cleavage and release of the LRP-ICD. It was also shown that LRP competes with APP for γ-secretase activity [125]. The LRP-ICD has been shown to translocate to the nucleus and interact with Fe65 and Tip60, although whether this occurs under physiological conditions remains to be determined. However, the recent observation that full length LRP1 levels were unaltered in BACE1^-/-^ mice, compared to wild type mice has shed doubt as to whether LRP is a true physiological substrate of BACE1 [126]. 

Neuroinflammation is a pathological feature of AD and increasing evidence suggests that neurotoxicity is mediated by CNS inflammatory processes whereby Aβ is involved in the activation of microglia facilitating the subsequent release of inflammatory cytokines including IL-1β, IL-6 and TNF-α, among others (reviewed in [127]). Interestingly, several putative BACE1 substrates are closely associated with the inflammatory response. 

P-selectin glycoprotein ligand 1 (PSGL-1) modulates leukocyte adhesion in inflammatory reactions and it was determined that cleavage of PSGL-1 by BACE1 liberated cleavage products observed *in vivo*, whereas no PSGL-1 cleavage fragments were detected in primary cells derived from BACE1 deficient mice [128]. A second membrane protein involved in regulating the immune response also appears to be a BACE1 substrate. BACE1 is enriched in neuronal Golgi membranes. Beta-galactoside alpha 2,6-sialyltransfe-rase (ST6Gal1) is a Golgi-resident sialyltransferase that is secreted out of the cell after proteolytic cleavage. BACE and ST6Gal I co-localize in the Golgi and BACE1 overexpression elevates ST6Gal I secretion [129]. Moreover, data from mouse models demonstrated that BACE1 cleaves ST6Gal I *in vivo* [130]. 

Ectodomain shedding is important in the inflammatory response. The interleukin-1 receptor II (IL-1R2) undergoes shedding and functions as a decoy receptor thought to limit the detrimental effects of IL-1 in the brain. Indeed, increased proteolytic processing and secretion of IL-1R2 has been linked to AD pathogenesis [131]. Recent data showed that IL-1R2 is processed by all three secretase moieties in a manner analogous to APP [132], and IL-1R2 secretion was elevated by BACE overexpression. Cleavage of IL-1R2 by both BACE enzymes occurred at sites that agreed well with the known cleavage specificity of BACE1 and BACE2 and led to the generation of CTF that were similar to those generated by α-secretase cleavage. It is noteworthy that in addition to IL-1R2, the processing of the type I membrane protein, TGFα and the GPI-anchored protein CD-16 was also elevated upon overexpression of the BACE enzymes. Interestingly, in contrast to other BACE substrates, the cleavage of IL-1R2 (and TGFα and CD-16) was not reduced in BACE^-/-^ cells. The authors argued against the fact that IL-1R2 may not be a physiological substrate of BACE by focusing on the BACE cleavage site of IL-1R2. BACE1 and BACE2 cleaved IL-1R2 only 4 amino acids away from the α-secretase cleavage site, so close that the α-secretase and BACE IL-1R2 CTFs would be very similar in size and likely impossible to resolve using electrophoresis. Thus, a decrease in BACE IL-1R2 cleavage would be compensated for by an increase in α-secretase cleavage and thus no net change in total IL-1R2 would be observed. Indeed, this is a valid point and, as the authors suggest, if BACE cleavage occurs close to the cleavage sites of specific substrates, such as TGFα and CD-16, by other proteases, it may be particularly challenging to unequivocally identify a specific protein as a novel BACE substrate. Thus in addition to IL-1R2, it may be the case that the BACE enzymes also have a more general role as alternative α-secretase-like proteases and are involved in the shedding of additional type I, II and GPI-anchored proteins known to undergo ectodomain shedding. In comparison to the large number of ADAM proteases, BACE1 and 2 are expressed at low levels. Thus if these proteases do act as alternative α-secretase moieties *in vivo*, their loss of expression could be compensated for by the ADAM proteases, which have partially redundant functions. Consequently, BACE knockouts, unlike ADAM protease knockouts, may exert more subtle, rather than striking, phenotypes, as demonstrated. 

The BACE1 substrates identified to date are all localized to the membrane and it has been previously suggested that BACE1 exerts a generalized role in the secretion of membrane proteins. However, Kuhn and colleagues investigated BACE cleavage specificity and observed that BACE did not cleave a number of membrane proteins including TNFα, P-selectin and CD14, suggesting that the proteases did not simply contribute to general membrane protein turnover [132]. 

## BACE1 INCREASES IN AD

Aβ plays a central role in AD pathogenesis. Aβ levels increase with age and excessive Aβ deposition occurs in AD. In FAD and DS, Aβ deposition can be attributed to excessive Aβ production mediated by APP/PS1 mutations or APP gene dosage effects. The mechanism by which excessive Aβ accumulation occurs in LOAD remains unclear. Reduced Aβ clearance and/or degradation is one potential mechanism leading to increased cerebral Aβ levels in AD. However, it is also possible that small increases in Aβ production over time may tip the balance toward Aβ accumulation. BACE1 is critical for Aβ biosynthesis and it is likely that factors that elevate BACE1 may lead to increased Aβ generation and promote AD. Indeed, FAD cases caused by the APP Swedish mutation, which enhances cleavage by BACE1, imply that increased BACE1 activity may be sufficient to induce AD pathogenesis. Furthermore, several recent reports have indicated that BACE1 dysregulation maybe involved in AD pathogenesis.

An age-related increase in BACE1 activity in mouse, monkey and non-demented human brain has been reported [133]. The increased activity appeared independent of brain region and although levels of BACE1 protein remain unchanged, there was a positive correlation between elevated BACE1 activity and increased Aβ levels in mouse and human brain regions. 

Furthermore, significant increases in BACE1 protein and activity have been observed in the AD brain. In high-order association brain regions affected by Aβ deposition BACE1 protein levels and activity were increased significantly in AD brain compared to non-demented control brain [134-138]. Given the competition for APP substrate, it is interesting to note that, in addition to increased BACE1 activity, a study demonstrated that in approximately two thirds of samples studied, α-secretase activity was decreased in AD temporal lobe tissue derived from LOAD patients [135]. Thus, eighty percent of AD brains examined had an increase in BACE1 activity, a decrease in α-secretase activity, or both [135].

Neuronal loss is a key feature of late-stage AD and thus, it is crucial that protein level and activity measurements are normalized to neuronal markers. Interestingly, the measures of BACE1 protein and activity levels were even more pronounced in AD brain when normalized to the synaptic marker, synaptophysin [134]. The significance of normalization to neuron-specific markers is further underscored by the findings of Harada that, in contrast to the above study, indicated that total BACE1 levels in AD temporal cortex was not increased [139]. However, the ratio of BACE1 protein to specific neuronal markers was significantly increased, indicating that the surviving neurons in AD brain may express higher BACE1 levels than those observed in neurons from control brain.

Although Fukumoto observed no significant correlation between elevated BACE activity and Aβ load in AD temporal cortex [134], measurements of total Aβ were taken rather than identifying the levels of specific Aβ species. However, Li and colleagues reported that the observed elevation of BACE activity is correlated with brain Aβ1-x and Aβ1-42 levels production in the frontal cortex [138], suggesting that indeed, BACE1 elevation may lead to enhanced Aβ production and deposition. Interestingly, a significant correlation between BACE1 levels and plaque load in AD brains was observed [137, 138].

BACE1 increases have also been observed in DS. People with DS inevitably develop characteristic AD neuropathology. However, APP gene dosage alone may not fully account for the AD pathology in DS [140]. Recently, a novel molecular mechanism by which AD may develop in DS has been proposed. Analysis of fetal DS and normal cerebral cortex indicated an elevation of C99, Aβ40 and Aβ42 in the trisomic tissue. The data indicated that the increase in APP level only partially contributed to Aβ over-generation in DS. Furthermore, the DS tissue showed a significant increase in the levels of total BACE1 protein, in particular mature BACE1 [141]. Importantly, subcellular fractionation of normal and DS brain tissue showed the marked accumulation of mature BACE1 in the Golgi fraction of DS cells. The authors proposed that the higher levels of mature BACE1 in DS tissue result in higher BACE1 activity leading to elevated C99 and Aβ production. The cause of the BACE1 elevation in DS remains to be determined.

Because aging is the strongest risk factor for AD, and BACE activity increases with age and to an even greater extent in LOAD, AD may reflect an exaggeration of age-related changes in BACE1 activity. Despite normalization of BACE1 levels to synaptic markers in AD brain, it remains difficult to determine from postmortem brain whether a specific change is an epiphenomenon in late-stage AD, or whether it is an early event directly involved in pathogenesis. Indeed, many biochemical parameters deviate from normal in AD and a recent proteomic study estimated that ~100 different proteins have deranged levels or abnormal modifications in AD (reviewed in [142]). To address whether the BACE1 elevation observed in AD brain is merely a passive end product of advanced neurodegeneration and cell death or whether it is actively involved in disease progression, we examined BACE1 levels in two Tg models of AD [143], namely the 5XFAD mouse [60] that develops amyloid plaques at young ages and exhibits significant neuronal loss, and the Tg2576 mouse [52], which develops plaques at older ages and does not show neuronal death. In contrast to human brain tissue, these Tg lines allow analysis of the effects of amyloid pathology on BACE1 elevation in the presence or absence of cell death [143]. BACE1 was elevated in the brains of both Tg models and AD patients. Importantly, because the BACE1 increase correlated with amyloid pathology in both Tg models and was observed in both the absence (Tg2576) and presence (5XFAD) of significant neuronal loss, we concluded that BACE1 elevation appeared to be associated with amyloid pathology rather than cell death. 

Many BACE1 antibodies are somewhat nonspecific [143]. Therefore, we generated a mono-specific BACE1 antibody, BACE-Cat1. BACE-Cat1-immunostaining showed the neuronal localization of BACE1, with BACE1 immunoreactivity surrounding Aβ42-containing plaque cores in both the Tg and AD brain. While further studies are required, the co-localization of BACE1 immunoreactivity with synaptophysin, but not MAP2, suggested a presynaptic localization for BACE1. Interestingly, an earlier study demonstrated similar findings [98] and our data suggest that the BACE1 elevation occurs in presynaptic neuronal structures around neuritic plaques and that Aβ42 may cause the increase. 

It is currently unclear whether the BACE1 elevation in AD promotes Aβ generation and disease progression. However, an AD feedback loop has long been surmised and our data are suggestive of a positive feedback loop, whereby Aβ42 deposition in AD causes BACE1 (and possibly APP) levels to rise in nearby neurons. Increased Aβ production may then ensue, initiating a vicious cycle of additional amyloid deposition followed by further elevated BACE1 levels. Given the elevation of BACE1 around Aβ42 plaque cores it seems possible that Aβ42 somehow triggers the BACE1 increase. It is well established that Aβ42 is neurotoxic and such toxicity may induce the BACE1 elevation. However, it remains undetermined as to which is the initiating event, Aβ elevation and deposition or increased BACE activity. Interestingly, a study recently proposed that Aβ acts *via *a biphasic neurotoxic mechanism, which is conformation-dependent, with Aβ oligomers inducing oxidative stress while fibrillar Aβ increases BACE1 expression and activity [144]. Nevertheless, it is currently unclear whether such a biphasic mode of action occurs *in vivo*.

## POTENTIAL TRIGGERS FOR BACE1 ALTERATIONS IN AD

AD pathogenesis is complex and its etiology undetermined. While risk factors for LOAD have been identified, the molecular mechanisms that link AD risk factors with the pathology of this disease are yet to be clarified. However, we do know that alterations in BACE1 (levels and/or activity) have been observed in AD and a number of risk factors, more over their downstream consequences, can be linked with such BACE1 alterations. 

AD and vascular diseases (both cardio- and cerebro-) appear to be intimately linked. Heart disease and stroke associate with elevated risk for AD, and have an established link with cerebral hypoperfusion (reviewed in [145]). Increasing evidence from neuroimaging [146-148] and epidemiological studies [149-152] suggests that vascular risk factors and the ensuing reduced cerebral blood flow (CBF) and chronic brain hypoperfusion (CBH) are key factors in AD development and may play a causative role in dementia. Indeed, CBH is a preclinical condition of mild cognitive impairment (MCI; a condition thought to precede AD) and is an accurate indicator for the prediction of AD development [153-157]. 

CBH can cause hypoxia, a form of cellular stress, together with ischemic episodes, which have been reported to increase AD risk [158, 159]. Indeed, oxidative stress has been implicated in AD pathology [160, 161] and defective energy metabolism may play a fundamental role in AD pathogenesis. Positron emission tomography imaging indicates that glucose utilization is lower in AD brain than in age-matched control brain [162-165], and MCI patients exhibit reduced glucose metabolism [166-168] suggesting that insufficient energy metabolism may be a factor in preclinical AD. Recently, data has emerged that links Aβ generation with cardiovascular disease. Importantly, the downstream consequences of vascular insults and the resulting CBH, including hypoxia, energy depletion and cellular stress appear to increase BACE1 levels and activity [169-175]. The potential mechanisms of how these cellular alterations could alter BACE1 are discussed below.

## PUTATIVE MOLECULAR MECHANISMS REGULATING BACE1 IN AD: THE COMPLEXITY OF BACE1 EXPRESSION

As just discussed, multiple recent studies have reported the elevation of BACE1 levels and/or activity in AD brain. Understanding the regulation of BACE1 expression may illuminate the role of BACE1 in normal biology, identify mechanisms that lead to disease, and might suggest approaches to inhibit BACE1 therapeutically. Here the regulation of BACE1 expression is discussed in detail. It is clear that BACE1 expression is regulated in a complex manner, both at the transcriptional and post-transcriptional level. We have used the latter phrase as an umbrella term to represent post-transcriptional processing and modification, mRNA stability, translation initiation, post-translational modifications and protein clearance. Thus, in addition to a number of transcriptional factors acting as either activators or repressors of BACE1 transcription, alternative splicing events, intracellular signal transduction pathways, post-translational modifications and alterations in protein half-life can affect the level and enzymatic activity of BACE1. Furthermore, BACE1 interacts with a number of other protein moieties, many of which are involved in protein trafficking, and so alterations in the level of these proteins may in turn affect BACE1 levels, subcellular localization and/or activity. It should be noted that the various AD risk factors discussed previously might impact BACE1 levels in a variety of ways, both at the transcriptional and post-transcriptional levels.

## TRANSCRIPTIONAL REGULATION OF BACE1 EXPRESSION

In order to gain insight into the regulation of BACE1 gene expression, both the human and rodent BACE1 gene promoters have been sequenced and analyzed [176-179]. Indeed, the BACE1 promoter is highly conserved between rodents and humans [176, 177] indicating that regulatory mechanisms of BACE1 expression are shared between these species and that rodents represent useful models in which to study mechanisms and to test therapeutics aimed at reducing BACE1 expression. 

The BACE1 gene promoter lacks the typical CAAT and TATA boxes, but contains six unique functional domains and three structural domains of increasing sequence complexity as the ATG start codon is approached [180]. It also contains a variety of transcription factor binding sites, including those for Sp1, GATA-1, AP1, AP2, CREB, estrogen and glucocorticoid receptors, NFκB, STAT1, YY1, HIF-1 and HSF-1, and PPARγ  [181] among others. It is likely that these sites influence transcriptional activity from the BACE1 promoter. 

The brain is particularly susceptible to hypoxic conditions and recent reports indicate that hypoxia can facilitate AD pathogenesis [173, 182]. During hypoxia, the hypoxia-inducible factor-1 (HIF-1) transcription factor binds to the hypoxia-responsive element (HRE) in target gene promoters and activates genes involved in energy metabolism and cell death [183]. Importantly, an HRE has been recently identified in the BACE1 gene promoter at base pairs -915-911, and quantitative RT-PCR demonstrated that mouse BACE1 mRNA expression, as observed for human BACE1 expression, appears to be upregulated by hypoxia at the level of gene transcription [173]. Indeed, the finding from *in vitro* assays indicating that hypoxia increased APP metabolism and Aβ production *via *BACE1 activity upregulation was confirmed *in vivo* [173]. 

Transient hypoxic insult to cortical neurons can cause mitochondrial dysfunction. While Aβ accumulation itself may lead to oxidative stress [144, 184], it has also been shown that oxidative stress may facilitate Aβ accumulation. 4hydroxy-2-nonenal (HNE), a marker of oxidative stress, has been identified at the early pathological stages of AD [160, 161]. Interestingly, oxidative stress *in vitro* resulted in significant increases in BACE1 promoter activity [171], and an increase in both BACE1 mRNA and protein levels, with increased BACE1 activity being observed following HNE exposure has been reported [169, 170].

Recent reports have documented BACE1 as a stress-induced protease [169, 170, 172, 174, 185-187], and in view of the apparent importance of metabolic dysfunction and amyloidosis in AD, it is worth noting that BACE1 upregulation has been observed under a variety of conditions including hypoxia, energy disruption and/or mitochondrial stress (discussed below). Further support for the role of BACE1 in response to stress comes from observations of BACE1 elevation following traumatic brain injury (TBI), following which, increased levels of BACE1 mRNA in hippocampal and cortical neurons were detected [186]. This increase was accompanied by a corresponding increase in BACE1 protein and activity. 

Neurons are responsible for the major portion of BACE1 and Aβ expression in the brain under normal conditions, and this is also likely to be true during AD. However, evidence is mounting that glia, and astrocytes in particular, may produce significant levels of BACE1 and Aβ, especially during inflammation. Indeed, a strong inflammatory reaction is present in AD brain and long-term use of nonsteroidal anti-inflammatory (NSAID) drugs reduces AD risk, suggesting inflammation may play an important role in AD pathophysiology [188]. 

Glia out-number neurons by ~10:1, so even a slight increase in glial BACE1 expression might contribute substantially to cerebral Aβ and exacerbate AD pathology. NFκB is increased in both aged and AD brain. Interestingly, Bourne [189] reported that NFκB acts as a repressor in neurons but as an activator of BACE1 transcription in activated astrocytes present in the CNS during chronic stress, a feature observed in AD. Furthermore, a functional NFκB site in the BACE1 promoter was stimulated in Aβ-exposed neural cells suggesting that elevated NFκB in the brain may significantly contribute to increased Aβ levels, acting as a positive feedback loop of chronic inflammation, astrocyte activation, and increased BACE1 transcription. Proinflammatory cytokines such as interferon γ  (IFNγ ), tumor necrosis factor α (TNFα) and interleukin β (IL1β) have been shown to increase Aβ secretion in cultured human astrocytes and astrocytic cell lines [190], and BACE1 levels rise, at least with IFNγ  treatment [191, 192]. Injection of INFγ  into mouse brain led to elevated astrocytic BACE1 expression. Molecular analysis indicated that INFγ  activated JAK2 and ERK1/2. Following phosphorylation, STAT-1 then binds to the putative STAT1 binding sequence in the BACE1 promoter region to modulate astrocytic BACE1 expression [192]. In some studies, reactive astrocytes around amyloid plaques appear to display BACE1 immunoreactivity in both Tg2576 Tg mice that develop amyloid pathology [193] and human AD brain [194-196]. However, subsequent analyses by our group on other APP Tg and AD brains show that plaques elevate BACE1 in neurons not astrocytes [143]. This discrepancy probably results from the use of BACE1 antibodies in the earlier reports that were not monospecific for BACE1, while our study used a novel, extensively validated BACE1 antibody that does not cross-react with other proteins. Although the BACE1 elevation in AD primarily occurs in neurons, the amplified number of astrocytes in AD brain is likely to result in a significant increase of astrocytic Aβ production.

Activation of PPARγ  with NSAIDs (e.g. ibuprofen) or PPARγ  agonists (e.g. pioglitazone) causes repression of BACE1 gene promoter activity, while proinflammatory cytokines that reduce PPARγ  levels lead to increased BACE1 mRNA [181]. Thus, the effects of inflammation and NSAIDs on AD may involve, at least in part, the action of PPARγ  on BACE1 gene expression. Interestingly, ibuprofen (and pioglitazone) treatment appeared to decrease BACE1 and reduce plaque load [197]. In the APP [V717I] Tg model of AD, BACE1 levels rose at focal sites of glial activation even before plaques begin to develop [198]. 

Taken as a whole, the evidence suggests that astrocytes may express significant levels of BACE1 and contribute to Aβ production, at least under certain proinflammatory conditions. In addition to inflammation other conditions may cause BACE1 expression to increase in the brain, including oxidative stress, TBI, hypoxia and ischemia. 

## THE POST-TRANSCRIPTIONAL REGULATION OF BACE1

Paradoxically, in some studies BACE1 mRNA levels in either neurons or astrocytes around amyloid plaques in APP transgenic brain are not elevated [69, 143, 199], implying that a post-transcriptional mechanism may be responsible for the BACE1 increase. Increased BACE1 protein and/or activity in the absence of altered BACE1 mRNA levels might be caused by several factors including post-transcriptional processing, a change in the rate of BACE1 translation and/or alterations in BACE1 protein stability.

The processing of pre-mRNA into multiple mRNA isoforms during alternative splicing is a powerful mechanism of genetic control. To date, four alternative splice variants of BACE1 have been identified and each encodes a protein isoform with different enzymatic activity [29, 30, 200-202]. Given the apparent elevation of BACE1 in AD brain, in the absence of BACE1 mRNA level elevation [134, 136], it is plausible that the differential expression of BACE1 isoforms may exert an effect on Aβ formation. BACE1 variants arise as a consequence of the alternative splicing of exon 3 and/or exon 4, producing an in frame deletion of 75 (25 amino acid deletion; BACE-I-476), 132 (44 amino acid deletion; BACE-I-457) and 207 (69 amino acid deletion; BACE-I-432) nucleotides. Reports have indicated that, unlike the enzymatically most active form BACE-I-501, both BACE-I-457 and BACE-I-476 lack β-secretase activity and are retained in the ER [29, 30]. While most human brains regions express all four BACE1 variants, the frontal cortex expresses high levels of BACE-I-501 and BACE-I-457 [200]. Interestingly, the same group examined the age-dependent differential expression of BACE1 and its four splice variants in six brain regions of young (4 months) and old (ten months) Tg2576 mice and compared them to wild-type litter mates [202]. The authors reported that BACE1 expression is regulated by age-dependent post-transcriptional regulatory mechanism in Tg2576 mice. While total BACE1 mRNA levels did not change in Tg2576 mice, the expression profiles of the BACE1 splice variants not only differed between Tg and non-Tg mice, but also changed with age. While Tg2576 mice expressed more BACE-I-501 more significantly than the other splice variants, wild type mice demonstrated a more balanced variant expression. However, during aging, wild-type mice undergo an increase in the relative abundance of the BACE1-I-501 variant. Young Tg2576 animals also expressed this BACE1 isoform at significantly higher levels as compared to age-matched wild-type mice. Given that Tg2576 mice overexpress human APP with the Swedish mutation, Zohar and colleagues speculated that it was this APP overexpression in Tg2576 mice that effected the differential expression of BACE1 splice variants in these animals and suggested that APP may exert a regulatory role on BACE1 transcripts. While these studies imply that splicing events are involved in the regulation of BACE1 enzymatic activity, the possible functions and brain distributions of BACE1 splice variants in AD brain remain to be established. As Zohar discusses, the differential expression of these variants may alter APP turnover and Aβ production [202].

Features of BACE1 5’ untranslated region (5’UTR) such as the GC content, the length, evolutionary conservation and the presence of upstream AUGs indicate that this 5’UTR may play an important role in the regulation of translational control and several studies indicate that BACE1 may be regulated in this fashion [203-205]. The use of BACE1 constructs devoid in part or all of the 5’UTR demonstrated that the presence of the 5’UTR could depress BACE1 translation significantly. Between the initiation site for transcription and the physiological translation initiation codon of the human BACE1 gene, there are 6 ATGs (uATGs) that could serve as translation initiation codons for five potential open reading frames (uORFs; [206]). The presence of such multiple upstream uORFs can inhibit the translation of the main ORF (reviewed in [207]). However, the mechanism by which the 5’UTR regulates BACE1 gene expression remains elusive. While Rogers proposed a shunting mechanism in which the ribosomal unit scanning along the transcript leader can jump and bypass ribonucleotide stretches [204], others [203, 205] favored the so-called leaky-scanning and re-initiation mechanism in which inhibition was mainly dependent on secondary structures and upstream AUGs. This hypothesis was supported by recent data that demonstrated that during human BACE1 gene expression, ribosomes skipped some uAUGs by leaky scanning and translated an upstream ORF initiated at the fourth uAUG. BACE1 translation was subsequently re-initiated at the physiological AUG site. It was hypothesized that alterations in the leaky scanning and re-initiation in BACE1 gene expression could be of importance in AD pathogenesis [206].

As previously indicated, increased BACE1 levels and activity have been reported in both *in vitro* and *in vivo* under conditions of altered energy metabolism and cellular stress. It is practically impossible to differentiate energy inhibition from oxidative stress with regards to the pharmacological blockage of mitochondrial energetic processes *in vivo* as mitochondrial respiratory inhibitors are generally considered to cause oxidative stress [208]. In agreement with previous studies indicating that energy inhibition is potentially amyloidogenic [169-171], following acute energy inhibition *in vivo* we observed a significant elevation in BACE1 protein that appeared to correspond with a significant increase in cerebral Aβ40 load in treated animals [172]. The mechanism of the energy-induced BACE1 increase *in vivo* is unclear and will likely be challenging to elucidate. While studies have demonstrated changes in BACE1 at the mRNA and protein levels following oxidative stress and hypoxic insults [170, 173], our data indicates that translational control may be implicated in the *in vivo* BACE1 elevation during times of energy depletion [172]. BACE1 mRNA levels did not significantly increase in Tg2576 brain following energy inhibition treatment and the BACE1 protein half life is too long to account for the rapid rise in BACE1 levels observed. As previously discussed reports indicate that the 5’UTR of BACE1 mRNA influences translational efficiency. Furthermore, the stress-activated p38 pathway has been shown to control translation through the 5’UTR and is likely elevated during energy metabolic stress. Interestingly, the HNE-dependent upregulation of BACE1 expression observed *in vitro*, appeared to be dependent on the activation of c-jun N-terminal kinases (JNK) and p38^MAPK ^[170]. Thus, we hypothesize that energy insufficiency, perhaps through p38, may elevate BACE1 mRNA translational efficiency *via *the 5’UTR. Clearly, further, detailed analysis is required to solve this important issue, but full understanding of such cellular mechanisms may shed light on novel therapeutic approaches for AD.

## BACE1 ACTIVITY: POST-TRANSCRIPTIONAL AND POST-TRANSLATIONAL MODIFICATIONS

The activity of the BACE1 enzyme is influenced by a number of factors, including splicing events, as previously discussed, the amount of BACE1 protein present (which is related directly to transcriptional and post-transcriptional events, along with altered BACE1 degradation), in addition to a variety of post-translational and cell biological events.

The activation of specific intracellular signaling molecules appears to be involved in the regulation of BACE1 expression in the brain. Evidence is emerging for the modulation of BACE1 expression by cholinergic receptor signaling although the data is somewhat conflicting and the level at which BACE1 is modulated (transcriptional verses post-transcriptional) remains unknown [209, 210]. Cholinergic dysfunction occurs early in the AD progression and many AD, FDA-approved therapeutics are aimed at improving cholinergic function [211]. While Zuchner reported that selective stimulation of M1 muscarininc acetylcholine receptors (mAChRs) increased BACE1 protein expression, with the activation of M2 mAChRs having opposing effects on the level of BACE1 protein [209], Caccamo and colleagues reported an impressive and almost complete inhibition of BACE1 protein expression following treatment of the 3xTg-AD model with a selective M1 muscarinic agonist [210]. While the reasons for these opposing data remain unknown, the reversal of the effect by a M1 antagonist was not investigated in the latter study, with the possibility that the observed effect on BACE1 levels may be due to mechanisms other than M1 receptor activation. Future studies should clarify this issue.

The subcellular trafficking and localization of BACE1 can impact the activity of this enzyme significantly. Hypercholesterolemia is a metabolic derangement that contributes to cardiovascular diseases, and epidemiological [212, 213], animal [214, 215], and cellular studies [216, 217] suggest that alterations in cholesterol homeostasis contribute to AD etiology by enhancing Aβ generation. During the exploration of the putative underlying molecular mechanisms of this apparent cholesterol-sensitive Aβ generation, Ghribi and colleagues reported that, in comparison to controls, cholesterol-fed rabbits exhibited elevated neuronal cholesterol levels and this cholesterol appeared to co-localize with BACE1 [218]. Furthermore, this association was accompanied by increases in both the level and activity of BACE1 and corresponding elevations in Aβ42. Such data indicate that the prevention of cholesterol accumulation, and/or cholesterol reduction may represent strategies for reduction of BACE1 over activation. Indeed, mature BACE1 localizes largely within cholesterol-rich lipid rafts [95, 96]; this localization may be enhanced by palmitoylation, and various lipids sti-mulate BACE1 activity [219]. Replacing the BACE1 transmembrane domain with a glycosylphosphatidylinositol (GPI) anchor exclusively targets BACE1 to lipid rafts and substantially increases Aβ production [220]. BACE1 is capable of forming stable homodimers that exhibit enhanced catalytic activity [221, 222]. Of interest are data that indicate that a decrease in cellular cholesterol levels by the cholesterol lowering statin drugs, prevented BACE1 palmitoylation *in vitro*, which consequently prevented BACE1 dimerization, facilitated a change in the subcellular location of the enzyme and a subsequent reduction in Aβ production [223, 224].

Interestingly, certain molecules have been shown to inhibit the BACE1-APP interaction and thus reduce β-site cleavage, including heparan sulfate [225], reticulon/nogo proteins [112], and sorLA/LR11 [226]. These may provide clues for designing strategies to inhibit BACE1 therapeutically. Other molecules have been shown to interact with BACE1 and increase enzyme activity, like prostate apoptosis response-4 (PAR-4) protein [227], while the effects of other interacting partners, such as the copper chaperone for superoxide dismutase-1 [228] and the brain-specific Type II membrane protein BRI3 [229], remain unclear. BACE1 and the γ-secretase complex are essential for Aβ genesis yet there is little data to support a functional relationship between BACE1 and the putative catalytic core of the γ-secretase complex. However, Kuzuya recently demonstrated that PS1 may promote BACE1 maturation [230]. Indeed, a role for PS1 in protein trafficking has been previously demonstrated [231, 232]. Interestingly, data indicates that the correct distribution of BACE1 is crucial for Aβ generation regulation, thus it is plausible that PS1 could somehow regulate BACE1 trafficking, thereby modulating its maturation.

Like APP, BACE1 cycles between compartments of the secretory pathway [233-235] and BACE1 activity resides in both the endosomes and secretory pathway. The intracellular trafficking and localization of the BACE1 protein is largely controlled by targeting signals present in the cytosolic portion of the C-terminal tail [235]. The DISLL sequence in the C-terminus of the BACE1 cytosolic tail (amino acids 496-500) is a so-called an acid cluster-dileucine motif (ACDL) and is known to be involved in endosomal trafficking. Deletion of the ACDL motif [233] or mutation of the leucines to alanines [235] alters the subcellular distribution of BACE1, such that a greater proportion of the protein is localized at the cell surface and less is sequestered within endosomes. 

The ACDL of BACE1 binds to members of the Golgi-localized γ-ear containing ADP ribosylation factor-binding (GGA) family implicated in the sorting of cargo proteins between TGN and endosomes [236-240]. GGA1, 2 and 3 are monomeric adaptors involved in transport of proteins containing the ACDL motif from the Golgi complex to the endosome, from endosomes to the TGN in the recycling pathway [239] and they may also be involved in the delivery of endosomal cargoes to the lysosome [241]. Koh and colleagues recently reported the lysosomal degradation of BACE1, and mutation of the di-leucine motif prevented lysosomal BACE1 accumulation following inhibition of lysosomal hydrolases [242]. Importantly, all three GGA proteins appear to be involved in the trafficking of BACE as depletion of any of the three through RNAi caused a significant BACE1 re-distribution [239]. GGA1 interacts with BACE1 and influences BACE1 trafficking through the recycling pathway. The ACDL motif interaction with GGA1 is modulated by serine phosphorylation of the BACE1 motif [234, 235, 237-239]. BACE1 phosphorylation at the S498 site and interaction with GGA proteins regulate the transport and recycling of the enzyme between early and late endosomes and the TGN. Whether dephosphorylation regulates BACE1 trafficking in other parts of the trafficking cycle remains to be determined. Although BACE1 phosphorylation does not appear to dramatically alter β-secretase activity in experimental systems, BACE1 trafficking may have a significant impact on Aβ production in the brain. 

Overexpression of GGA1 led to an increase in the levels of both immature BACE1 and APP species [243]. Despite the immature status of BACE1, APP metabolism still occurred and elevations in C99 and APPsβ were observed. However, levels of Aβ were reduced, data indicative that GGA1 blocked APP β-cleavage products from becoming γ-secretase substrates. It was demonstrated that GGA1 confined APP to the Golgi [243]. Thus not only does GGA1 interact with BACE1, but it acts also as a sorting protein that affects APP trafficking and ultimately the proteolysis of this molecule [243, 244]. Interestingly, a very recent report has highlighted the individual roles of the GGA proteins in mediating BACE1 trafficking. In contrast to GGA1, Tesco and colleagues demonstrated that an inhibition of GGA3, *via *RNAi, led to an elevation of BACE, C99 and Aβ [185]. 

BACE1 can be degraded by at least three mechanisms: 1. endoproteolysis within its catalytic domain [245]; 2. the ubiquitin-proteasomal pathway [246]; 3. the lysosomal path-way [242]. Evidence for altered BACE1 degradation first came from Puglielli and colleagues who reported that the lipid second messenger, ceremide, increases BACE1 half-life and the generation of Aβ [247]. Interestingly, the same group reported that treatment of neuroblastoma cells expressing the p75 neurotrophin receptor (p75^NTR^) with nerve growth factor, elevates both ceremide and BACE1 levels [248]. The authors demonstrated a link with the aging process, the single most important risk factor for AD, whereby normal aging activates Aβ generation in the brain by “switching” from the tyrosine kinase receptor A system to that mediated by p75^NTR^. Indeed the Puglielli group have just reported that ceremide stimulates the carrier-mediated translocation of acety-CoA into the lumen of the ER, a prerequisite for the lysine acetylation of BACE1 (detailed previously) that is necessary for BACE1 maturation and stabilization [92]. 

Occlusion of the middle cerebral artery is a widely accepted experimental stroke model used to study the effects of transient cerebral ischemia. Not only did ischemia lead to an elevation in BACE1 protein and activity but data from co-localization experiments indicated that BACE1 immunoreactivity was strongly associated with TUNEL staining, a marker of apoptosis [187]. Interestingly, a recent study has revealed a potential molecular mechanism that may underlie the BACE1 increase following ischemic episodes [185]. Whilst it is appears that ischemia induces apoptosis, the contribution of apoptosis to AD pathogenesis remains unclear although there is increasing evidence for caspase activation in the AD brain (reviewed in [249, 250]). However, apoptosis enhances Aβ levels in both neuronal and non-neuronal populations and in the recent study from Tesco and colleagues [185], BACE1 levels and associated activity were potentiated during apoptosis. Indeed, caspase activation during programmed cell death induced the BACE1 increase *via *a post-translational stabilization of BACE1 and a significant impairment in BACE1 degradation and turnover. As previously detailed, the GGA adaptor proteins are implicated in the subcellular trafficking of BACE1. Tesco demonstrated that GGA3 is cleaved by activated caspase-3 during apoptosis. In the rat ischemia model, this reduction in GGA3 levels was co-ordinated with caspase activation and increased BACE1 protein levels. Furthermore, RNAi silencing of GGA3 caused an increase in the level and activity of BACE1 as determined by elevations in C99 and Aβ. Indeed, He *et al*. have previously demonstrated that RNAi-mediated depletion of GGAs significantly increases endosomal BACE1 levels [239]. Degradation of BACE1 occurs, in part at least, in the lysosomal pathway [242] and a role for GGA3 in the targeting of cargo to the lysosome has been previously reported [241]. Thus, Tesco and colleagues suggested that apoptosis, caused by ischemic events, drives GGA3 depletion and results in the stabilization and accumulation of BACE1 leading to elevated enzymatic activity. Importantly, in AD brain, GGA3 protein levels were significantly decreased and in AD-relevant regions, this decrease was inversely correlated with elevations in BACE1 [185]. While other post-translational mechanisms may account for this observed decrease in GGA3, it is tempting to speculate that apoptotic events may play a role in the increase in BACE1 in AD brain, indeed, elevated BACE1 activity may lead to increased Aβ levels and, given that Aβ can induce apoptosis, this could potentially trigger a vicious cycle that self-potentiates Aβ generation and cell death.

## CONCLUDING REMARKS

Almost a decade since the initial identification of BACE1 as the β-secretase our understanding of this enzyme continues to increase. BACE1 is a clear drug target for inhibiting Aβ production, and it is highly likely that a partial inhibition of BACE1 activity may be beneficial, although the percentage of BACE1 inhibition required to significantly delay amyloid pathology and the associated cognitive changes requires further determination.

While the physiological role (s) of BACE1 remain to be conclusively determined, indications are that BACE1 may function as a stress response protein. Not only are several recently identified BACE1 substrates involved in the response to stress and/or injury such as axonal growth and the regulation of glial cell survival (NRG1; [251]), recovery from excitotoxicity (Aβ; [106]), Aβ clearance (LRP; [252]), synapse formation (APP, APLP1, APLP2; [253, 254]), neuroprotection (secreted APP ectodomain; reviewed in [255]) and immune functions (PSGL-1, ST6Gal I and IL-1R2; [128, 129, 132]), but BACE1 levels are elevated during stressful conditions, including those related to vascular disease [134, 141, 143, 169-174, 218]. We suggest that the elevation in BACE1 levels facilitates the recovery after acute stress/injury and it is plausible that cleavage of BACE1 substrates is necessary for this function.

However, it remains a possibility that chronic stress/injury results in pathologic BACE1 levels and deleterious amyloid formation. Indeed, the elevation in BACE1 observed in AD may provide a molecular link between known AD risk factors (such as vascular disease and injury) and AD pathogenesis, and the molecular mechanisms governing these associations are being intensely investigated. Interestingly, vascular disease and its downstream consequences, together with TBI, have been linked to alterations in BACE1 on a variety of levels, both transcriptional and post-transcriptional. Where as TBI, hypoxia, oxidative stress, and inflammation may affect levels of BACE1 mRNA [169-171, 173, 181, 182], energy deficiency and metabolic stress might impact the translation of BACE1 [172]. BACE1 activity is also affected by the microenvironment, being stimulated by various lipid moieties [219], and the stability of BACE1 may be affected by ischemic conditions [185]. Clearly further understanding of the molecular mechanisms of BACE1 elevation during AD may accelerate the development of novel therapeutic strategies to treat this neurodegenerative disease.

## ABBREVIATIONS

AD: = Alzheimer’s disease
Aβ: = β-amyloid
APP: = Amyloid precursor protein
BACE1: = β-site APP Cleaving Enzyme 1
FAD: = Familial AD
PS: = Presenilin
DS: = Down’s syndrome
LOAD: = Late onset AD
ApoE: = Apolipoprotein E
CTF: = Carboxyl terminal fragment
AICD: = APP intracellular domain
TACE: = TNF-α converting enzyme
ADAM: = A disintegrin and metalloprotease domain protein
TGN: = Trans Golgi network
BACE1^-/-^: = BACE1 knockout
Tg: = Transgenic
SNP: = Single nucleotide polymorphism
ER: = Endoplasmic reticulum
PC: = Proprotein convertase
CSF: = Cerebral spinal fluid
APLP: = Amyloid precursor-like proteins
NRG1: = Neuregulin 1
PNS: = Peripheral nervous system
CNS: = Central nervous system
VGSC: = Voltage-gated sodium channel
RIP: = Regulated intramembrane proteolysis
LRP: = Lipoprotein receptor-related protein
ICD: = Intracellular domain
PSGL-1: = P-selectin glycoprotein ligand 1
ST6Gal1: = Beta-galactoside alpha 2,6-sialyltransferase
IL-1R2: = Interleukin-1 receptor II
CBF: = Cerebral blood flow
CBH: = Chronic brain hypoperfusion
MCI: = Mild cognitive impairment
HIF-1: = Hypoxia-inducible factor 1
HRE: = Hypoxia-responsive element
HNE: = 4hydroxy-2-nonenal
TBI: = Traumatic brain injury
NSAIDS: = Nonsteroidal anti-inflammatory drugs
PPARγ: = Transcriptional regulator proliferator-activated receptor γ
INFγ: = Interferon γ
TNFα: = Tumor necrosis factor α
IL1β: = Interleukin β
UTR: = Untranslated region
uORFs: = Open reading frames
mAChR: = Muscarinic acetylcholine receptor
GPI: = Glycosylphosphatidylinositol
PAR-4: = Prostate apoptosis response-4
ACDL: = Acid cluster-dileucine motif
GGA: = Golgi-localized γ-ear containing ADP ribosylation factor-binding
RNAi: = RNA interference
EGF: = Epidermal growth factor
PI3K: = Phospatidylinositol-3-OH kinase
JNK: = c-jun N-terminal kinases
P75^NTR^: = p75 neurotrophin receptor
